# Comparative Analysis of Medical School and Physician Assistant Education and the Use of Provisional Licenses

**DOI:** 10.7759/cureus.72969

**Published:** 2024-11-04

**Authors:** Manu M Mathew, Samantha O'Connor, Tisha M Titus

**Affiliations:** 1 Family Medicine, American University of Antigua, New York, USA; 2 Hospital Medicine and Emergency Medicine, Daemen University, Amherst, USA; 3 Family and Preventive Medicine, Emory University, Atlanta, USA

**Keywords:** assistant physician, pance, physician assistant, physician shortage, usmle exams

## Abstract

The current shortage of physicians in the United States has prompted a reevaluation of medical education and licensure pathways. This paper analyzes medical school and physician assistant education, highlighting the differences in training, clinical experience, and licensing requirements. Additionally, the paper explores the emerging role of postgraduate licensure (PGL) programs, which allow unmatched medical graduates to obtain provisional licenses and practice under attending physician supervision. Through this analysis, we assert that medical graduates are underutilized assets in addressing the healthcare workforce deficit with PGL programs as a viable solution to alleviate physician shortages, particularly in primary care.

## Introduction and background

The current shortage of physicians in America has prompted a reevaluation of the existing medical education system. The insufficiency of residency slots, despite the increasing number of medical school graduates, exacerbates this scarcity [1].

The Association of American Medical Colleges (AAMC) has provided annual updates on the ongoing physician shortage with statistics from 2021 recognized by legislators as problematic, resulting in the introduction of the Resident Physician Shortage Reduction Act in 2023. If passed, this would create an additional 2,000 residency positions annually for seven years, totaling 14,000 additional residency positions [2].

Even if this bill is passed, the persistent residency shortage, the expansion of medical schools, and the existing physician shortage will still result in an insufficient number of trained physicians to meet healthcare demands. This is especially apparent within the high-demand field of primary care, due to an aging population [3]. Despite a 28% increase in the number of board-certified internal medicine and family medicine physicians from 2012 to 2022, demand remains unmet, and there is a projected shortage of 35,260 primary care physicians by the year 2035 [4,5].

Additional attempts to combat the physician shortage have included the increased utilization of non-physician practitioners, such as physician assistants (PAs). PAs are healthcare practitioners educated at a master’s level. Per the Physician Assistant Education Association (PAEA), “PAs work together with doctors as part of an integrated medical team” to provide high-quality patient care [6].

The 2024 AAMC report demonstrates little to no improvement in the estimated physician shortage, especially in primary care specialties [3]. In response, novel approaches like the Assistant Physician Program in Missouri have emerged. These programs allow unmatched medical graduates to obtain limited licensure and work under physician supervision, similar to the current practice model of PAs.

With nearly 2,000 unmatched medical school graduates annually, they are a viable working resource that is underutilized throughout America [7]. The medical school curriculum is more comprehensive in depth and breadth of topics than the PA curriculum and requires significantly more testing and hands-on supervised clinical care hours. Given this, medical school graduates are more qualified and able than new graduate PAs to provide safe, high-quality care under the supervision of an attending physician.

## Review

Physician education, training, and licensure

Physician Education and Training

The medical school curriculum spans four years and is typically divided into pre-clinical and clinical years called the 2 plus 2 model, which includes 5,000 to 6,000 supervised clinical care hours, multiple Observed Structured Clinical Exams, and National Board of Medical Examiners shelf exams after every clinical rotation. Curriculums transitioned to the New Pathway in 1987 with the introduction of problem-based learning and clinical time throughout. Core pre-clinical science subjects include anatomy, physiology, pathology, pharmacology, immunology, embryology, biostatistics, epidemiology, microbiology, and behavioral science. Clinical rotations include pediatrics, surgery, obstetrics and gynecology, internal medicine, psychiatry, neurology, family medicine, emergency medicine, acting internships, dedicated licensure study time, research, electives, and a capstone research project [8].

Physician Licensing Process

The United States Medical Licensing Exam (USMLE) is a three-step, four-day exam taken across allopathic medical schools and residency that culminates in the ability to apply for licensure to practice medicine; however, without completion of a residency and board certification, there are few employment options. The Comprehensive Osteopathic Medical Licensing Examination of the United States is a similar process for osteopathic medical schools, with some students electing to also take the USMLE to afford more residency options.

Step 1 is an eight-hour test taken during the second year of medical school consisting of seven 60-minute blocks of up to 40 questions covering the application of basic sciences to medicine [9]. Step 2 CK is a nine-hour test taken during the third year of medical school consisting of eight 60-minute blocks of up to 40 questions covering the ability to apply medical knowledge, skills, and understanding of clinical science essential for providing supervised patient care. Step 3 is a two-day test typically taken during the first year of residency. Day one is a seven-hour test consisting of six 60-minute blocks of up to 40 questions covering the foundations of independent practice. Day two is a nine-hour test consisting of six 45-minute question blocks and 13 computer-based case simulations covering advanced clinical medicine. While Steps 1 and 2 can be taken in any order, Step 3 must be taken last and after completion of a Doctor of Medicine (MD) or Doctor of Osteopathic Medicine (DO) degree.

If any step in the process is attempted four or more times, including incomplete attempts, without a passing score, the candidate becomes ineligible to apply for any other USMLE exams. There is a maximum of three attempts on any step in a 12-month period, and passed steps are not allowed to be retaken. Many states place a seven-year limitation on the total time allowed to complete all three steps, but this is state-dependent. Passing the USMLE steps makes one eligible to apply for licensure, with each state determining its own requirements for initial medical licensure.

Residency Training and Board Certification

After completion of medical school and two of the three USMLE step exams, residents will typically obtain a training license but must complete Step 3 and the state-required residency time to be eligible for full licensure. Most hospitals and insurers require physicians to be board-eligible or board-certified to hire them and bill for patient care.

Residency consists of graduated responsibility under supervision, which progresses from direct to indirect over three to five years, which varies by specialty. After completion of residency, additional years of fellowship may be completed with an additional board exam lasting one to two days. Each board exam consists of a written exam, with or without an additional oral exam, that confers diplomate (board-certified) status.

The American Board of Medical Specialties (ABMS) is currently composed of 24 boards, 40 specialties, and 89 subspecialties. In 2023, there were 988,000 board-certified physicians [4], which is approximately 89% of the 1.1 million practicing physicians in the US [10].

Physician Financial Burden

The cost of attending medical school has increased by 20% since 2009, with the most recent numbers from 2019 indicating a median total cost of $272,000 [11]. While there are some scholarships and grants, the majority of students will take out federal or private loans with a fixed interest rate of 6.5-7.5%. This is often added to existing undergraduate loans that average $22,200 to $44,600 [12].

The current average resident salary is $64,000 per year starting in PGY1, with increases every subsequent year of $2,000 to $5,000, with considerable variability in salaries depending on specialty, location, and program [13]. Residents have the option to pay off their student loan debt during this time, or they may seek grace periods, deferment, or forbearance [14].

The average physician salary after residency is $243,000 for generalists and $346,000 for specialists [13]. Depending on the type of loan and repayment strategy, loan repayment can take 10 to 30 years [15].

PA education, training, and licensure

PA Education and Training

PA programs average 27 months in length and include didactic and clinical portions designed to train and educate students for supervised clinical practice [16]. The didactic portion of PA programs consists of anatomy, physiology, biochemistry, pharmacology, physical diagnosis, pathophysiology, microbiology, clinical laboratory science, behavioral science, and medical ethics [17].

Clinical rotations occur over 11 months and require PA students to rotate through specialties including family medicine, internal medicine, obstetrics and gynecology, pediatrics, general surgery, emergency medicine, and psychiatry [16]. Successful completion of each rotation is determined by the hours met, satisfactory evaluations by the preceptor, and a passing grade on the PAEA End of Rotation exam. During clinical rotations, PA students complete approximately 2,000 supervised clinical care hours [17]. PA students are also required to learn how to search, interpret, and evaluate medical literature, often as part of a research project [16].

PA Licensing Process

In order to apply for licensure, PA school graduates must pass the Physician Assistant National Certifying Examination (PANCE), which is a single exam designed to assess the fundamental medical knowledge and clinical intervention abilities pertinent to PA practice [18,19]. Many states offer an interim one- to two-year license for those who have not passed the PANCE, allowing them to work without certification [20].

The exam consists of 300 multiple-choice questions that are administered in five separate blocks. Each 60-minute block consists of 60 questions. The PANCE has two focus areas: knowledge of the diseases and disorders PAs encounter and knowledge and skills related to the tasks PAs perform when treating patients. These tasks primarily involve obtaining a history and physical examination, ordering and interpreting diagnostic studies, and formulating a diagnosis and treatment plan [21].

Graduates may take the PANCE up to six times within a six-year period. If either the number of attempts or the six-year period is exhausted, graduates are deemed ineligible to take the PANCE. In order to regain eligibility, individuals must again complete a PA program [21].

Once licensure is obtained, PAs are able to directly enter into supervised clinical practice, and the type and amount of supervision a PA receives are determined at the state level and again at the practice site. Depending on where a PA holds licensure, the type of supervision a PA receives may include strict chart review and co-signature requirements, direct supervision, remote supervision, collaboration without supervision, or no supervision [22]. Any additional training and education that is required for specialties are completed on the job, and the ability to work in such specialties does not require the completion of a residency, fellowship, or any other additional educational training.

PA Financial Burden

The most recent values from 2019 indicate that the median student loan debt for PA school graduates is $112,500, and this is in addition to the cost of undergraduate loans [23]. The majority of students take out federal or private loans with a fixed interest rate.

According to the 2023 AAPA Salary Report, the median annual compensation for a PA is $127,000 [24]. Depending on the payment plan, the length of time needed to repay student debt ranges from 10 to 25 years [23].

Postgraduate Licensure (PGL) Programs

In light of the physician shortage crisis, PGL programs have been developed as a novel approach to ease the physician shortage. PGL licensure allows medical graduates who have been unable to secure a residency position to practice under the supervision of attending physicians. These programs address physician scarcity by integrating graduates into the healthcare system. Additionally, PGL programs reduce healthcare costs related to physician shortages and provide unmatched graduates with valuable clinical experience and professional growth, which enhances their skills and confidence in clinical settings.

Benefits of PGL Licensure

PGL programs offer substantial economic benefits by being more cost-effective than hiring attending physicians, nurse practitioners, or PAs, who typically command higher salaries. PGLs reduce costs and barriers associated with physician shortages, such as increased patient wait times and reliance on expensive temporary staffing. These programs also help healthcare facilities maintain high-quality care while managing costs effectively.

Until the residency shortage can be resolved, PGL programs support the professional development of medical graduates by offering them valuable clinical experience under the supervision of expert physicians. This hands-on experience builds confidence and competency in clinical settings, improves their chances of a successful match in subsequent residency application cycles, and helps to strengthen the pipeline of physicians in training with limited educational disruption. Additionally, these programs provide unmatched medical graduates with the opportunity to earn an income, which helps alleviate their educational debt and maintain financially functional households. While specific data on their compensation is limited, anecdotal evidence suggests that assistant physicians in Missouri typically earn between $0 and $20 per hour. The salary is primarily determined at the discretion of the supervising physician and/or the employing organization.

Barriers to PGL Licensure

PGLs have time restrictions on renewals, often forcing PGL holders to re-register or acquire residency status within a set number of years. For example, in Missouri, PGLs are renewable for up to three years, after which the licensee must either match into a residency or stop practicing. There are misconceptions regarding the duties of PGLs, with the prevailing belief being that they are not clinically competent or able to work under attending supervision and that this may result in a reduced standard of care.

Generally, PGLs are not able to bill insurance, nor are they able to use incident to billing, which is the same for resident interns working under a training permit. Anecdotally, there are concerns that PGLs are poorly compensated compared to PA graduates, which has led to concerns that PGLs may be misused as low-cost labor in low-value positions. The temporary nature of PGL programs is a significant barrier, as it may lead to concerns about stability and reliance on temporary staffing.

A recent court case in New York, where a medical school graduate was denied a PA license, highlights the complexities in creating alternative licensure pathways. The ruling emphasized the need for distinct qualifications between professions, reinforcing that any new pathway must carefully align with professional standards [25].

Comparison analysis

**Figure 1:**
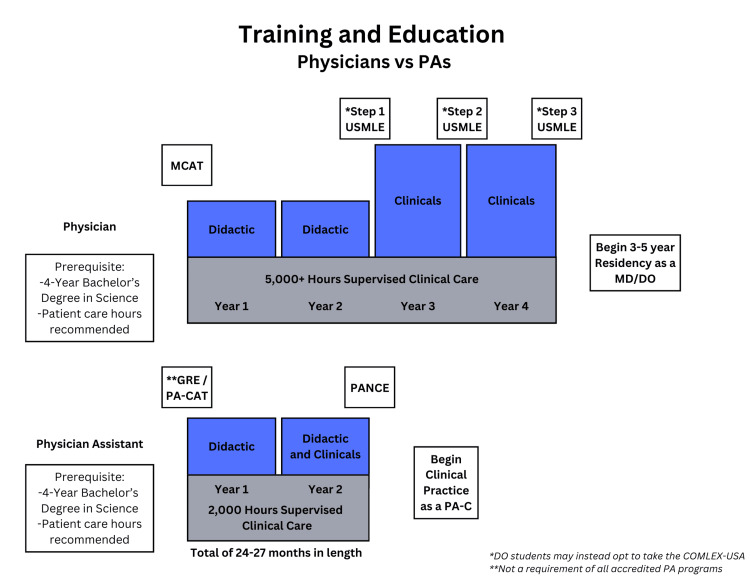
Training and education physicians vs. PAs PA, physician assistant

PAs are medical providers who are trained in the medical model (a science-based physiology and pathophysiology methodology for diagnosis, prevention, and treatment of medical problems) to work under the supervision of a physician. Given this, the education and training that a PA receives is shorter and less comprehensive than that of a physician who is specifically training to practice independently. Compared to PAs, medical students complete almost twice as much schooling, obtain over twice as many supervised clinical care hours, and have to pass four times as many examinations to be eligible for licensure, as demonstrated in Figure 1. Given that medical school and the USMLE are more lengthy and comprehensive, medical school graduates who pass the first 2 steps of the USMLE are more qualified than new graduate PAs to obtain licensure to practice medicine under the supervision of an attending physician. This extensive education and rigorous testing ensure that medical graduates possess a broader and deeper understanding of clinical conditions, making them well-prepared to deliver high-quality patient care.

PGL programs are an effective solution to utilize the skills of unmatched medical graduates who represent a ready and able workforce to help combat the physician shortage, and this would be in line with the interim licensure that PAs are allowed to obtain without passing their certification exam. Every year, thousands of medical graduates do not secure a residency, primarily due to a lack of available positions, resulting in a significant underutilization of trained professionals. By integrating PGLs into the healthcare system, these graduates can provide essential medical services under the supervision of an attending physician, such as preventive screenings, school and work physicals, and the management of chronic medical conditions like hypertension, diabetes, and high cholesterol, which addresses both healthcare workforce shortages and disparities in healthcare access (Table 1).

**Table 1:** PGL programs by state/US territories and description The following programs have been passed, allowing medical graduates to practice under the supervision of a board-certified physician while seeking residency positions. The ones that have been implemented are indicated with an asterisk (*). PA, physician assistant; PGL, postgraduate licensure

Program	States/US territories	Description
Assistant physician	Missouri* and Florida*	Allows unmatched medical graduates to work under supervision while seeking residency positions [26,27]
Associate physician	Utah*	Allows unmatched medical graduates to work under supervision in clinical settings while seeking residency [28]
Bridge physicians	Louisiana*, Idaho, and Alabama*	Transitional role for unmatched medical graduates reapplying for residency, enabling them to gain clinical experience [29-31]
Graduate physician	Tennessee	Allows unmatched medical graduates to work in clinical settings while seeking residency positions [32]
Graduate registered physician	Arkansas*	Allows unmatched medical graduates to work under supervision while seeking residency positions [33]
House physician	Florida*	Unmatched medical graduates practice under the direct supervision of an MD or DO in-training program [34]
International medical graduate clinical experience license	Washington*	Provides unmatched international medical graduates with clinical experience under supervision [35]
Limited permit	New York* and Arizona*	Permits unmatched medical graduates to work in specific clinical roles under supervision [36,37]
PA	Commonwealth of Puerto Rico*	Allows unmatched medical graduates to work as a PA with a medical degree [38]
Special permit for graduates of the University of Kansas School of Medicine	Kansas*	Permits unmatched medical graduates to work under the supervision of a licensed attending physician [39]
Supervised medical graduates	Maryland	Allows unmatched medical graduates to gain supervised clinical experience [40]

In a selected interview conducted on individuals who completed both MD and PA education, training, and licensing requirements, Kristin Tott, MD FACS, stated, “I felt very comfortable with continuing to learn under direct supervision of my surgeon MD after PA school, but not solo. After MD training, I would’ve felt the same without specialized residency training.”

## Conclusions

We acknowledge the limitations of the lack of comparative data, and this is a readily identified area of needed research. Moving forward, ideally, there would be consistent nomenclature, licensure pathways, and performance requirements for PGLs, as well as data supporting the quality and outcomes of care they provide.

PGL programs present an innovative solution to mitigate the physician shortage by integrating unmatched medical graduates into clinical practice under supervision. By enabling these graduates to provide essential services, such as managing chronic conditions and conducting physicals, PGL programs offer a practical pathway to address both workforce shortages and health access disparities. The successful implementation of these programs will be critical to bridging the gap in the healthcare system.
